# A novel mutation in COL1A1 causing osteogenesis imperfecta/hearing loss

**DOI:** 10.1016/j.bjorl.2023.101312

**Published:** 2023-08-25

**Authors:** Ti-Ti Pan, Lin Han, Hong-Wei Zheng, Zhi-Min Xing, Li-Sheng Yu, Yuan-Jun Liu

**Affiliations:** Peking University People’s Hospital, Department of Otorhinolaryngology, Head and Neck Surgery, Beijing, China

**Keywords:** Osteogenesis imperfecta, Hearing loss, Stapes surgery

## Abstract

**Objectives:**

To screen the COL1A1 and COL1A2 gene mutation sites in a family with type I osteogenesis imperfecta (OI)/hearing loss and analyze the characteristics and recovery of hearing loss in patients with osteogenesis imperfecta.

**Methods:**

The basic clinical data of OI proband and her parents were collected, and the COL1A1 and COL1A2 genes were detected in peripheral blood by PCR amplification and generation Sanger sequencing. Literature of stapedial surgery in patients with osteogenesis imperfecta was collected.

**Results:**

The heterozygous mutation of the 26 exon c.1922_1923 ins C in the OI progenitor COL1A1 gene led to the amino acid frameshift mutation of p.Pro 601FS, which was not detected in the phenotypic parents. The homozygous of exon 28 c.1782>G in COL1A2 was detected in the proband and her parents, resulting in changes in the protein p.Pro 549Ala.

**Conclusion:**

The clinical symptoms of the OI proband is caused by heterozygous mutation of the 26 exon c.1922_1923 ins C in COL1A1 gene. Stapedial surgery can provide short-term and long-term hearing benefits for OI patients with hearing loss.

**Level of evidence:**

Level 4.

## Highlights

• We report a novel mutation in COL1A1 causing osteogenesis imperfecta/hearing loss.
• Stapes surgery leads a significant reduction in hearing threshold and air-bone gap.
• Patients can obtain short- and long-term benefit from the stapes surgery.

## Introduction

Osteogenesis imperfecta (OI) is a rare genetically heterogeneous connective tissue disorder, with a prevalence of 1/15,000–20,000.^1^ OI is characterized by increased bone fragility, decreased bone mineral density, and skeletal deformities, and the clinical symptoms are characterized by frequent bone fractures, blue sclera, hearing loss, laxity of joint and skin, short stature, and dentine. OI has an autosomal dominant or recessive inheritance pattern of inheritance and has been found to be associated with at least 20 genes, including thousands of mutation types. More than 90% of OI patients are due to mutations in the genes encoding collagen type 1 (COL1A1, COL1A2).^2^ Type 1 collagen is the main protein in bone and other connective tissues, and consists of two α1 and one α2 chains, which form a “glycine-X-Y” triple helix structure. Changes in the structure or number of triple helices caused during gene transcription, translation, and protein modification can lead to symptoms of varying severity.^3^ Among them, COL1A1 is located at 17q21.33 includes 51 exons encoding pre-α1. The OI database (The COL1A1 gene homepage, https://databases.lovd.nl/shared/genes/COL1A1) has reported hundreds of mutations in the COL1A1 gene and the types of the gene mutations are still being enriched.

Studies have shown that patients with OI can be accompanied with progressive Hearing Loss (HL), which mostly occurs in the second to fourth decade of life and that about 50% of OI patients have hearing impairment at the age of 50, while patients with normal hearing at the age of 50 are unlikely to develop hearing impairment thereafter.^4^ However, it has also been suggested that the proportion of patients with HL approaches 100% with increasing age. Pathologic examination of the temporal bone (including the oval window) undergoes pathological changes similar to those seen in otosclerosis, with the lack of ossification, decalcification, but not identical, and may be accompanied with microfracture of the acoustic tuberosity, fixation of stapes footplate and occlusion of the round window. Thus, HL can be conductive, sensorineural or mixed.^5^, ^6^, ^7^, ^8^

Studies related to HL in patients with OI are highly limited due to clinical heterogeneity. Therefore, we identified a novel mutation locus in COL1A1 by genetic testing of OI patients and summarized the current status and prognosis of hearing loss studies in OI patients, with the aim of providing some help in the diagnosis and treatment of OI patients with hearing loss.

## Methods

### Patients

The proband, a 38-year-old woman who complained with progressive right hearing loss and tinnitus for 30 years and her parents (non-inbreeding) were brought into the study. Clinical characteristics and laboratory examinations were collected, including height, weight, scleral color, hearing, tooth condition, fracture history, bone mineral density, X-ray of the fracture site, 25-(OH) D_3_, PTH, thyroid function, gonadal six items (Follicle-producing hormone, luteinizing hormone, estradiol, progesterone, testosterone, prolactin), blood calcium and phosphorus.

### Gene test

Peripheral blood Deoxyribonucleic Acid (DNA) samples were collected, and DNA was extracted by DNA Kit from TIANGEN BIOTHECH (DP348), all exons of COL1A1 and COL1A2 were sequenced by Polymerase Chain Reaction (PCR) amplification and generation Sanger sequencing using the kit. The results were compared with the public database, and the pathogenicity was tested by Mutation Taster, Polyphen2 and SIFT online prediction software.

## Results

**Figure 1:**
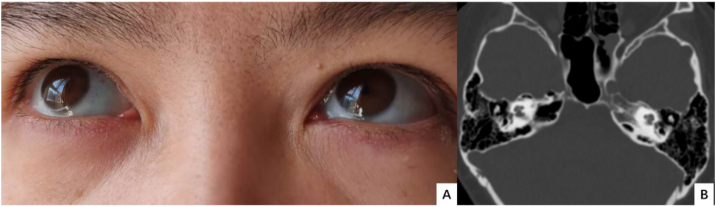
(A) Bilateral blue sclera of the proband; (B) CT scan of the temporal bone is normal.

**Figure 2:**
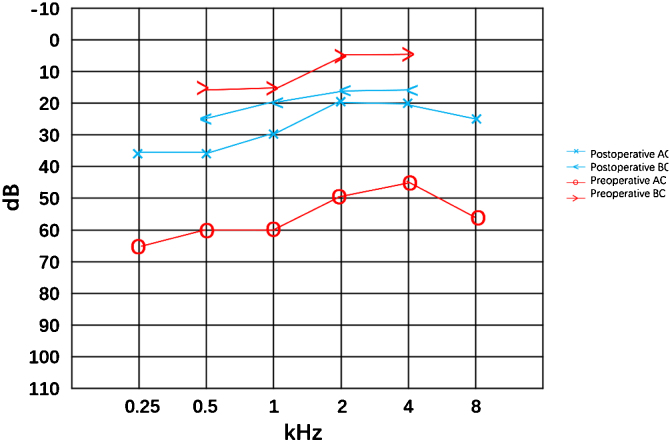
Conductive hearing loss preoperative with a gap of 45 dB and the gap decreased to 8 dB postoperative.

The proband suffered from asthma, high blood pressure, multiple cavities, and recurrent limb fracture for three times previously. Physical examination found bilateral blue sclera (Fig. 1), the normal appearance of bilateral tympanic membrane and that she was 170 cm tall with a Body Mass Index (BMI) of 20.76 kg/m². Rinne test was negative and weber test was lateralized to the right ear. The Computerized Tomography (CT) scan showed nothing was abnormal in the middle ear (Fig. 1). The preoperative pure tone showed conductive hearing loss of the right era with a gap of 45 dB (Fig. 2). Conductive hearing loss combined with OI was suspected. Relevant abnormal laboratory tests and examinations were collected (Table 1). Imaging examinations showed degenerative changes of the knee joint, cervical spine, and lumbar spine.

**Table 1:** Abnormal examinations and tests of the proband.

	Observed value	Reference range
Bone mineral density (SD)	−2.3	−1.0 to −2.5
Ultrasound pachymetry (μm)	453	500–600
Total N-terminal propeptide of type 1 collagen (ng/mL)	14.25	15.13–58.59
25-(OH) D3 (nmoL/L)	20.27	Deficiency: <50
PTH (pg/mL)	75.41	15.00–65.00
Thyroglobulin antibody (IU/mL)	74.0	<60
Thyroid peroxidase antibody (IU/mL)	227.8	<60
Serum calcium (mmoL/L)	2.09	2.20–2.65

A stapes surgery with otomicroscope under general anesthesia was performed. The soft Incudomalleolar joint, fixed stapes foot plate and slender stapes crura were found intraoperatively which was the same to the previous findings. The incudostapedial joint as well as the anterior and posterior arches of the stapes were disconnected, and the Piston was implanted to reconstruct the ossicular chain. Postoperative hearing (8 months after the surgery) was significantly improved. The air-bone gap was decreased from 45 dB to 8 dB. There was a slight increase in the sensorineural threshold after the surgery, which was thought be the natural progress or the measuring error.

### Gene test and mutation analysis

**Figure 3:**
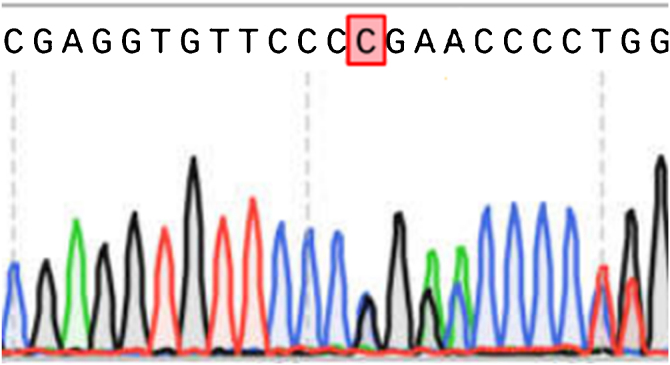
Results of COL1A1 exon sequencing (reference sequence: NM000088).

**Figure 4:**
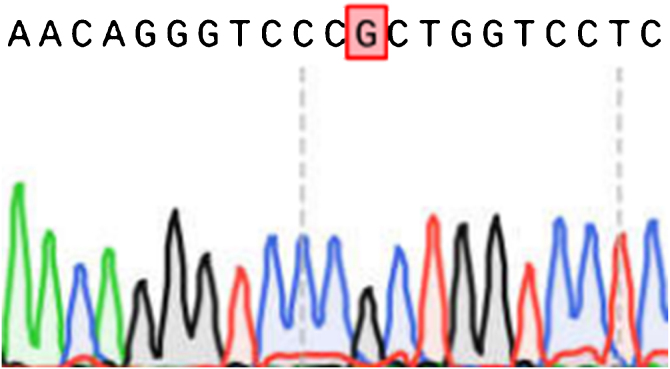
Results of COL1A2 exon sequencing (reference sequence: NM000089).

Peripheral blood samples were collected from the proband and her parents for DNA detection of all exons of COL1A1 and COL1A2 by PCR amplification and Sanger sequencing by kit (DIA-UP Biotech, Beijing), and NM000088 and NM000089 were used as reference sequences respectively. The results showed that the proband had a heterozygous mutation of c.1922_1923 ins C in exon 26 of the COL1A1 gene, leading to the change of p.Pro 601 fs protein (Fig. 3), which was predicted to be “disease causing” by Mutation Taster. In addition, a homozygous mutation of c.1782>G in exon 28 of the COL1A2 gene was detected in all three patients resulting in the change of p.Pro 549 ala protein (Fig. 4). Polyphen2 and SIFT were used for functional prediction of c.1782>G mutation, which indicated that this mutation was neutral and less likely to cause disease.

## Discussion

We report a novel missense mutation c.1922_1923 insC p.Pro 601 fs in COL1A1 in an OI proband, which is not present in the asymptomatic parents. The mutation was predicted to be pathogenic. This frameship-mutation is of great significance, since the first 601 amino acids are fixed, but the subsequent amino acids have changed and may generate a new reading frame, thus expressing a completely different protein. 1782>Gp.Pro 549 ala homozygous mutation of COL1A2 is found in all three subjects. Given that the parents have no clinical symptoms of OI and the mutation site is predicted to be “neutral”. Therefore, we believe that the clinical symptoms of OI proband may not be related to the c.1782>Gp.Pro 549 ala mutation in exon 28 of COL1A2, but the c1922incC p.Pro 601 fs mutation in exon 26 of COL1A1. This discovery enriches the gene pool of congenital osteogenesis imperfecta and provides a scientific basis for prenatal diagnosis.

There have been a large number of clinical studies on the epidemiology, type, severity and treatment of hearing loss in OI patients, but there is no unified conclusion at present, and the treatment is to reconstruct the sound transmission and sensory system by surgery. It is generally believed that HL in OI patients occur mostly in the second to fourth decade of life and tends to stabilize after the age of 50.^4^, ^9^, ^10^ It is often bilateral, can be asymmetric, and its prevalence depends on the type of OI, most common in type I and least in type IV.^11^ It can be conductive, mixed, and sensorineural. Conductive is the main form in young people, while sensorineural is more common in elderly people.^12^ It has been believed that HL may gradually develop from conductive to mixed or sensorineural with increasing age.^13^ Mean hearing threshold increased by 0.5 dB (4 kHz) to 1.7 dB (8 kHz)/year in patients with type I OI between 10 and 45 years, with sensorineural accounting for 0.6 dB (-0-4.5 k-Hz) to 1.3 dB (8 kHz)/year and conductive accounting for 0.4 dB (full frequency)/year.^14^ It has also been suggested that patients with lower BMD are more likely to develop conductive fractures, which may be related to the fact that patients with this type are more likely to accumulate microfractures and interfere with the inhibitory pathway of bone remodeling in the temporal bone, leading to stapes foot plate fixation.^15^ The occurrence, type and severity of HL are not related to the nature of OI gene mutation, and there is obvious intra-family heterogeneity.^16^

Most patients with ABG can benefit from the stapes surgery. Fixation, thickening, easy fragility of the stapes foot plate, fracture, and atrophy of the stapes crura, bleeding and thickening of the middle ear mucosa, and atresia of the oval window can be found in the temporal bone of OI/hearing loss (Table 2). This patient underwent stapes surgery in otomicroscope and we found the incudomalleolar joint soft, the stapes footplate fixed and the stapes crura slender same to the previous reports. Relevant reports on hearing gain after stapes surgery in patients with OI/hearing loss is presented in Table 3 from the Pubmed database. Patients may obtain better air conduction hearing benefits in the short and long term after stapes surgery. There has about 30 dB hearing gain of AC in this patient in 8 months postoperatively. However, the appearance of sensorineural hearing loss components may aggravate the hearing loss in the long-term follow-up process after surgery. Cochlear implantation is an option for severe/profound sensorineural.

**Table 2:** Intraoperative findings in stapes surgery of OI.

Intraoperative findings	Stapes footplate (%)			Stapes crura (%)		Mucosa (%)	
	Fixed	Thick/brittle	Thin	Fracture	Atrophic	Excessive bleeding	Thick
Kuurila et al.^17^	/	19	4	4	4	16	/
Hijazi et al.^18^	18 (100)	9 (50)	/	5 (27)	n	2 (11)	/
Swinnen et al.^19^	29 (100)	22 (76)	/	1 (3)	13 (45)	6 (21)	/
Doi et al.^20^	14 (100)	6 (43)	/	8 (57)	8 (57)	/	/
Van der Rijt and Cremers^21^	13 (100)	7 (54)	/	1 (8)		9 (69)	6 (46)
Dieler et al.^22^	7 (88)	/	6 (75)	4 (50)	/	/	/
Skarzynski et al.^23^	(56)		(26)	/		(15)	/
Swinnen et al.^24^	13 (100)	/	/	1 (8)	4 (31)	2 (15)	5 (39)
Pedersen and Elbrond^25^	43 (100)	23 (53)	/	5 (12)	14 (33)	13 (30)	12 (28)
Pedersen and Elbrønd^26^	11 (100)	5 (45)	/	1 (9)	9 (82)	4 (36)	3 (27)
Garretsen and Cremers^27^	54 (93)	32 (55)	/	10 (17)	22 (38)	12 (21)	7 (12)
Shea and Postma^28^	62 (100)	/	/	/	13 (21)	18 (29)	/
Cremers and Garretsen^29^	14 (100)	10 (71)	/	3 (21)	3 (21)	6 (43)	/

**Table 3:** Results of short-term and long-term of ABG and HG following stapes surgery.

Author	n (ears)	Femal:male	Mean age	Mean time of follow-up (y)	ST HG ≥ 10 dB		LT HG ≥ 10 dB		ST ABG (<10 dB)		LT ABG (<10 dB)		HG of mean AC threshold (dB)	
					No	%	No	%	No	%	No	%	ST	LT
Garretsen and Cremers^13^	58	31:18	30.6	9.6	49	85	27	68	37	71	26	70	24	16
Kuurila et al.^17^	43	9:24	30.1	5.6	30	70	/	/	18	42	/	/	/	/
Hijazi et al.^18^	18	9:2	37.5	4	13	76	12	92	9	53	6	46	18.4	22.4
Swinnen et al.^19^	34	13:9	32.7	16.2	26	93	17	90	17	61	18	95	24.3	27.7
Doi et al.^20^	15	8:3	32.6	6.5	/	/	/	/	13	93	/	/	/	/
Swinnen et al.^24^	15	6:6	40	3.5	12	92	8	89	8	67	6	75	25.9	25.8
Vincent et al.^30^	46	14:11	36	2.7	/	/	/	/	17	94	13	72	19.7	17.9
Vincent et al.^31^	23	14:4	37	/	/	/	/	/	18	86	8	80	20	19
Shea and Postma^28^	51	29:14	/	7	43	84	16	67	38	75	/	/	30	/
van der Rijt and Cremers^21^	13	7:6	38	3.6	11	85	/	/	4	31	/	/	27	/

Short-term (ST) hearing gain: ≤12 months; long-term (LT) hearing gain: >1 year; HG, hearing gain; HL, hearing loss; ABG, air-bone gap; AC, air conduction; BC, bone conduction.

## Conclusion

Our study finds a novel mutation site c.1922_1923 ins C in COL1A1 causing osteogenesis imperfecta/hearing loss. Stapes surgery leads to a significant improvement in hearing thresholds and reduction in ABG. Patients with hearing loss in OI can obtain short-term and long-term benefit from the stapes surgery.

## Funding

This research received no specific grant from any funding agency in the public, commercial, or not-for-profit sectors.

## Ethical approval

This study was approved by the Administration Committee of Peking University People’s Hospital, China (2021-278).

## Statement of human rights

Ethical approval to report this case was obtained from the Administration Committee of Peking University People’s Hospital, China.

## Statement of informed consent

Written informed consent was obtained from the patients for their anonymized information to be published in this article.

## Conflicts of interest

The authors declare no conflicts of interest.
